# Emphysematous cystitis following a transrectal needle guided biopsy of the prostate

**DOI:** 10.1186/1471-2334-12-322

**Published:** 2012-11-26

**Authors:** Takeshi Hashimoto, Kazunori Namiki, Ayako Tanaka, Kenji Shimodaira, Tatsuo Gondo, Masaaki Tachibana

**Affiliations:** 1 Department of Urology, Tokyo Medical University, 6-7-1 Nishishinjuku, Shinjuku-ku, Tokyo 160-0023, Japan

## Abstract

**Background:**

Emphysematous cystitis (EC) is a comparatively rare urinary tract infection characterized by air within the bladder wall and lumen and is usually associated with immunosuppression or poorly controlled diabetes mellitus.

**Case presentation:**

We report a case of EC in a 70-year-old man who recently underwent transrectal ultrasound needle-guided prostate biopsy, after which he underwent pylorogastrectomy. He did not have any history of diabetes mellitus or any immunosuppressive disease. The patient developed severe sepsis, requiring intravenous antibiotics and urinary catheterization. Despite therapy, the patient developed disseminated intravascular coagulopathy and acute respiratory distress syndrome. Therefore, he was admitted to the intensive care unit, antibiotic coverage was broadened, and danaparoid sodium and sivelestat sodium hydrate was administered. After 20 days, the patient’s condition improved, and on the 28th day, the patient was discharged to home in a good condition without any sequelae.

**Conclusion:**

Prompt diagnosis and treatment are warranted to prevent potential morbidity of and mortality in cases of EC.

## Background

Emphysematous cystitis (EC) is the presence of intramural gas along with or without luminal gas within the bladder caused by primary infection of the lower urinary tract in a gas-producing organism. The disease is most common among middle-aged diabetic women and is relatively rare in patients who do not have immunosuppressive disease.

## Case presentation

A 70-year-old man, who did not have any history of diabetes mellitus, long-term steroid use, abnormalities in the immune system, or long-term urinary catheter use and a normal digital rectal examination result, had elevated serum prostatic specific antigen (PSA) level of 13.3 ng/mL. Because of the elevated serum PSA level and stomach cancer, the patient underwent a transrectal ultrasound needle-guided prostate biopsy and a pylorogastrectomy on the same day. Before the procedures, the patient received a rectal enema and an oral fluoroquinolone antibiotic.

**Figure 1:**
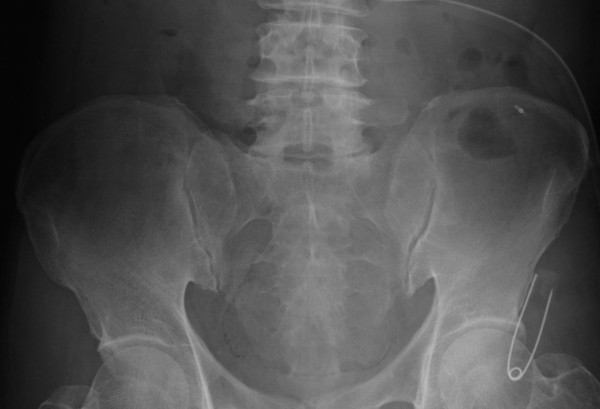
Plain radiograph of the abdomen showing a nonspecific gas pattern and a small amount of air in the true pelvis, in the shape of the bladder.

The transrectal ultrasonography indicated a gland weighing 60 g without any abnormalities. The patient underwent an uneventful 12-core prostate biopsy. However, after 4 days, the patient developed a fever (body temperature, 101°F) associated with gross hematuria. The patient was noted to have a white blood cell count of 10500 cells/mm^3^, a hemoglobin level of 9.7 g/dL, a platelet count of 17.8 × 10^4^/mm^3^, and a C-reactive protein level of 14.8 mg/dL. The urinalysis result demonstrated more than 50 white blood cells/h.p.f. Urine and blood culture specimens were obtained. On a bladder scan, a post-void residual urine volume of 350 mL was noted. Acute prostatitis was diagnosed, and intravenous meropenem therapy was initiated. Despite the administration of the antibiotic, the patient’s body temperature was still 104°F. Subsequently, a plain radiograph of the abdomen and a computed tomography (CT) image of the abdomen and pelvis (with and without intravenous contrast) were obtained. The plain radiograph of the abdomen revealed a nonspecific gas pattern and a small amount of air in the true pelvis in the shape of the bladder (Figure 1).

**Figure 2:**
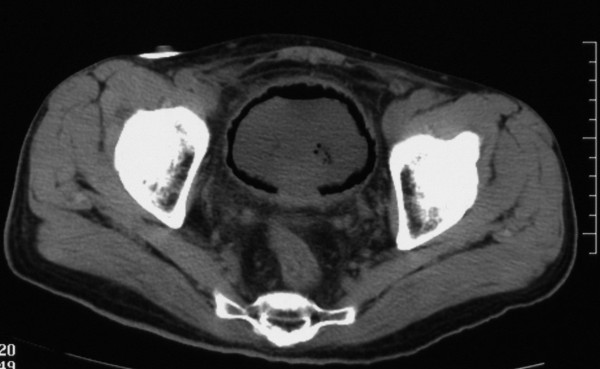
Computed tomography image showing extensive gas within the wall of the urinary bladder.

The diagnosis of EC was confirmed on the CT image, which revealed the presence of extensive gas within the wall of the urinary bladder (Figure 2).

The blood and urine cultures were positive for pan-sensitive *Escherichia coli*. A urinary catheter was then inserted for draining the bladder. Despite these maneuvers, the patient developed disseminated intravascular coagulopathy (DIC) and acute respiratory distress syndrome, requiring transfer and monitoring in the intensive care unit. The patient’s antibiotic treatment was changed from ciprofloxacin to vancomycin. In addition to the standard supportive procedures, patient therapeutic regimen of danaparoid sodium and sivelestat sodium hydrate was initiated. After 20 days of conservative management, the patient’s condition improved, and on hospital day 28, the patient was discharged to home in a stable condition. After 1 year, the patient did not report any sequelae from the EC.

## Discussion

EC is a relatively uncommon condition. Of the cases of EC, 60% are associated with diabetes mellitus [1,2]. Kelesidis et al. [3] reported that EC was commonly observed in patients who had either diabetes or other immunosuppressive diseases. Moreover, they reported that the other risk factors were old age, female sex, urinary tract outlet obstruction, structural abnormalities of the bladder, chronic urinary tract infection, hemorrhagic cystitis, and alcoholic disease.

In the present case, EC developed secondarily after major surgery and prostate biopsy. Kimura et al. [4] reported that surgical and traumatic injuries profoundly affect innate and adaptive immune responses and that a significant suppression in cell-mediated immunity after an excessive inflammatory response appears to be responsible for the increased susceptibility to subsequent sepsis. This report might indicate that the immune system of the patient weakens with surgery, in a similar manner observed in immunosuppressive disease. In our recent review of the literature, some of the reported cases of EC developed secondarily from medical intervention (Table 1).

**Table 1:** Emphysematous cystitis occurred secondary to medical intervention

**Author**	**Year**	**Age**	**Gender**	**Diabetes mellitus**	**Medical interference**	**Complication**	**Treatment (except antibiotics)**	**Culture**	**Prognosis**
**Kalra O.P.**	**1993**	**35**	**M**	**-**	**renal transplant**	**chronic glomerulonephritis**	**graft nephrectomy**	***Klebsiella pneumoniae***	**died**
**Dimitris D.**	**1996**	**59**	**M**	**+**	**orchiectomy**	**epididymitis**	**surgical debridement cystotomy**	***E. coli***	**recovered**
**Anwar N.**	**2002**	**57**	**F**	**+**	**peritoneal dialysis**	**hypertension, chronic kidney disease**		***E. coli***	**recovered**
**Van G. E.**	**2004**	**50**	**F**	**No data**	**hysterectomy**	**vesicovaginal fistula**	**intensive care, urinary diversion**	***Klebsiella pneumoniae enterococcus faecalis***	**died**
**Thomas A.**	**2006**	**80**	**M**	**-**	**colectomy**	**colitis**	**urinary drainage**	***E. coli***	**recovered**
**Yokoo T.**	**2007**	**68**	**F**	**+**	**hemodialysis**	**hypertension, chronic kidney disease**		***E. coli***	**recovered**
**Carla M.**	**2008**	**81**	**M**	**-**	**replace the mitral valve**	**myocardial infraction**	**intensive care**	***Enterobacter aerogenes***	**died**
**Mok H.P.**	**2010**	**80**	**F**	**No data**	**anterior resection of rectum**	**data not reported**		***E. coli***	**recovered**
**Sereno M.**	**2010**	**70**	**F**	**+**	**chemotherapy**	**breast cancer**		***E. coli***	**recovered**
**Lang E.K.**	**2011**	**44**	**M**	**No data**	**renal transplant**	**hypertension, chronic kidney disease**	**surgical debridement, nephrostomy**	**No data**	**not reported**
**present case**	**2012**	**70**	**M**	**-**	**gastrectomy, prostate biopsy**	**benign prostatic hypertrophy**	**urinary drainage**	***E. coli***	**recovered**

*E.coli: Escherichia coli.*

Patients with EC might have varied clinical presentations, ranging from incidental diagnosis on abdominal imaging to severe sepsis [5]. Symptoms and signs include stomach pain, irritative voiding symptoms, hematuria, and pyuria. Pneumaturia is also a characteristic symptom of EC but requires differentiation from vesicoenteric fistula and vesicovaginal fistula [1,2,6,7]. EC was first reported in a canine in 1926. The occurrence of EC in a human was first reported in 1961 [1]. Improved imaging techniques over the past several decades have allowed increased detection of EC. Plain radiography of the abdomen has a sensitivity of 95% in detecting air within the bladder wall. However, CT is needed to obtain a differential diagnosis of EC and to rule out vesicoenteric fistula or vesicovaginal fistula. It is feasible that magnetic resonance imaging and ultrasonography may prove to be valuable diagnostic tools for EC [7,8].

The mechanism of aerosis in EC is still unknown. The bacterium responsible for approximately 58–60% of the reported EC cases is *E. coli*, followed by *Klebsiella pneumoniae*, accounting for approximately 18–21% of the cases [1,6,7]. The other bacteria found to be associated with EC include *Proteus*, *Staphylococcus*, and *Clostridium*. It is thought that these organisms can ferment glucose present in urine, producing acid and CO_2_. The transportation of accumulated CO_2_ away from the tissue is disrupted by local inflammation or obstructive uropathy. The accumulation of gas further increases the local pressure and may lead to infarction of the adjacent tissues, augmenting the barrier to gas transportation and thus creating a vicious cycle [6].

Treatment of EC consists of broad-spectrum antibiotics and placement of a urinary catheter to drain the bladder. In our case, we hesitated to insert the urethral catheter because acute prostatitis is believed to lead to EC and insertion of a urethral catheter was generally not preferred for acute prostatitis. Furthermore, we were also uncertain whether a cystostomy should be performed, as it might not be suitable in cases with an infectious bladder. With ingravescent course, the patient needed to undergo urine drainage because of residual urine. We, then, decided to insert a urethral catheter because he rejected cystostomy. If a patient has diabetes mellitus, then immediate blood glucose control should be warranted. In severe cases, septicemia may lead to DIC, which occurred in our patient. The death rate from EC is reported to be approximately 7–10%, illustrating the need for rapid diagnosis and early treatment [1,6,9].

## Conclusions

We report a case of EC occurring after a transrectal needle-guided biopsy of the prostate. The patient did not have any history of diabetes mellitus and urinary tract infection. However, in addition to the transrectal biopsy, stress by pylorogastrectomy was thought to cause the adverse event. Successful management of EC depends on its early diagnosis, with correction of the underlying causes and administration of the appropriate antibiotics. Early detection and prompt treatment are encouraged.

## Consent

Written informed consent for publication of this case report and any accompanying images was obtained from the parents. A copy of the written consent is available for review by the editor-in-chief of this Journal.

## Abbreviations

DIC: Disseminated intravascular coagulopathy; EC: Emphysematous cystitis; CT: Computed tomography; PSA: Prostatic specific antigen.

## Competing interests

The authors declare that they have no financial or non-financial competing interests.

## Authors’ contributions

TH and KN drafted the manuscript. TH, KN, AT, KS and TG were the attending physicians throughout the disease course. MT is the Chairman and the Professor of Department of Urology. All authors read and approved the final manuscript.

## Pre-publication history

The pre-publication history for this paper can be accessed here:

http://www.biomedcentral.com/1471-2334/12/322/prepub
